# Supplementary figures and images for: A widespread family of heat-resistant obscure (Hero) proteins protect against protein instability and aggregation
Source: PLoS Biol. 2020 Mar 12;18(3):e3000632. doi: 10.1371/journal.pbio.3000632 (PMC7067378; doi:10.1371/journal.pbio.3000632)

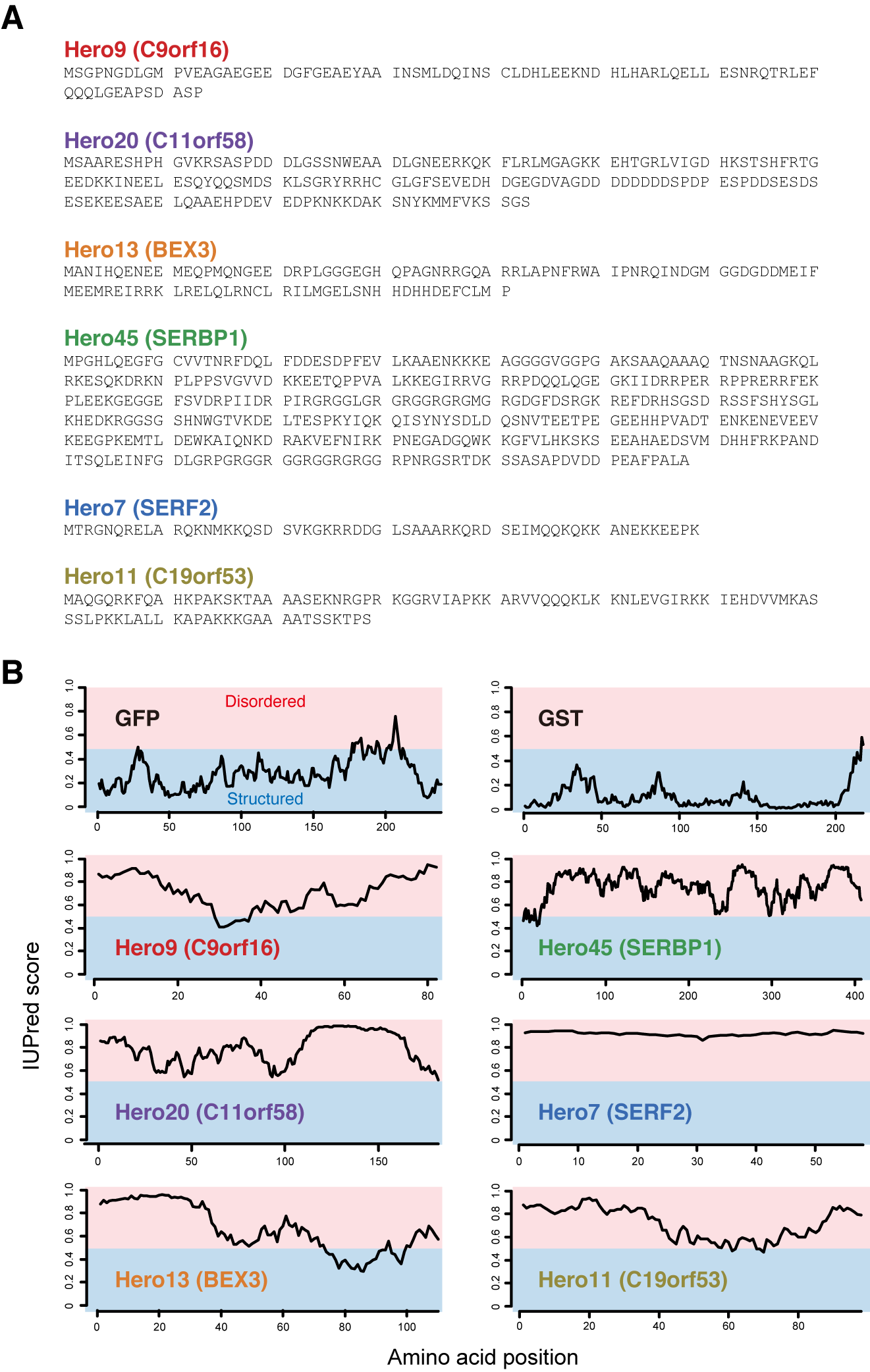

Supplement: S1 Fig — (A) Amino acid sequences of the six representative Hero proteins. (B) Disorder prediction by IUPred using the default settings [16]. GFP and GST were used as controls. GFP, green fluorescent protein; GST, glutathione S-transferase; Hero, heat-resistant obscure. (TIF) [file pbio.3000632.s001.tif]

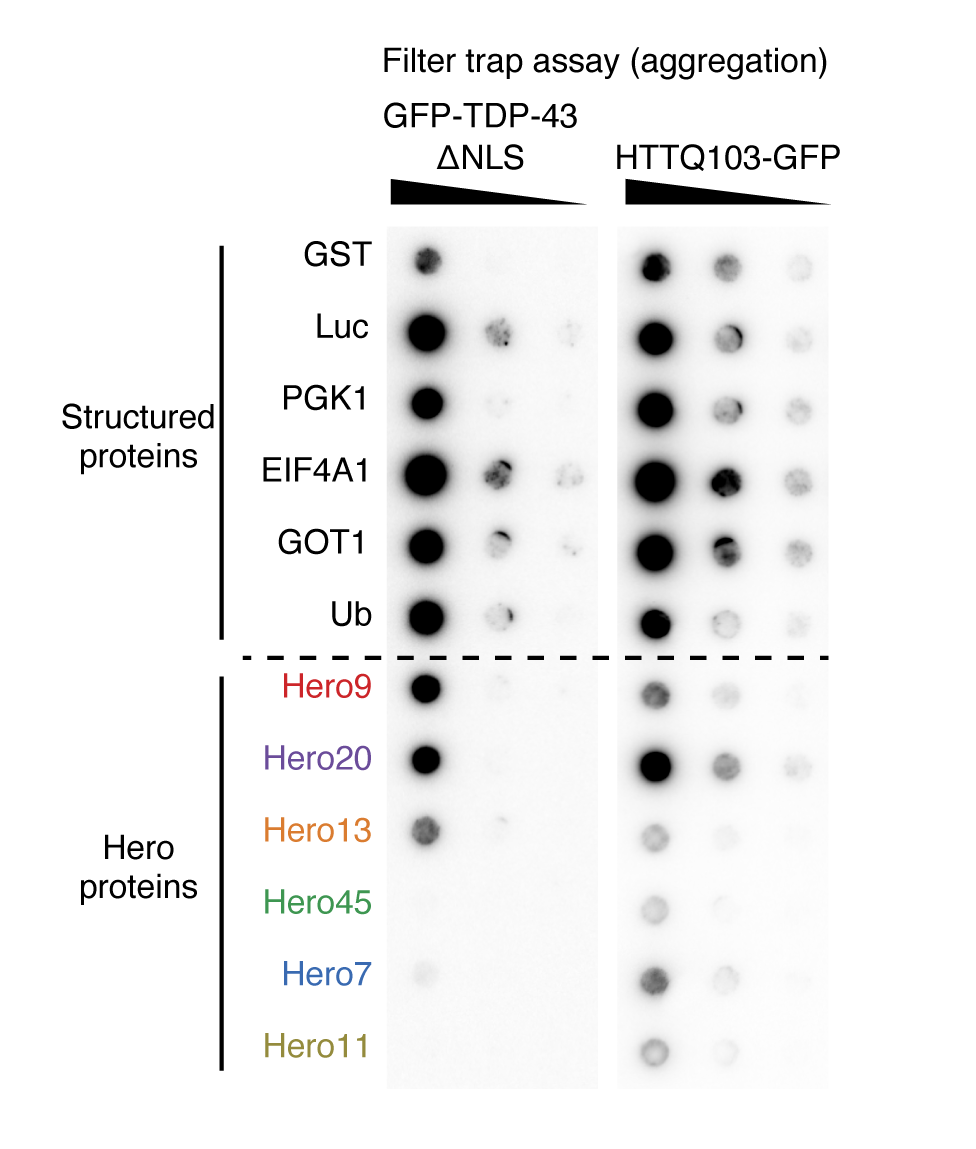

Supplement: S2 Fig — Filter trap assay of aggregation-prone proteins expressed in cells. TDP-43ΔNLS or HTTQ103 was expressed in a GFP-fusion form in HEK293T cells, together with each Hero protein, GST, or other structured proteins. The original or 5-fold diluted samples were loaded on a cellulose acetate membrane in the presence of 1% SDS, and the trapped aggregates were probed with anti-GFP antibody. We observed the same effects in two independent experiments. GFP, green fluorescent protein; GST, glutathione S-transferase; Hero, heat-resistant obscure; TDP-43ΔNLS, TDP-43 lacking the nuclear localization signal. (TIF) [file pbio.3000632.s002.tif]
