# Supplementary material for: A widespread family of heat-resistant obscure (Hero) proteins protect against protein instability and aggregation
Source: PLoS Biol. 2020 Mar 12;18(3):e3000632. doi: 10.1371/journal.pbio.3000632 (PMC7067378; doi:10.1371/journal.pbio.3000632)

# Fig.1B raw image

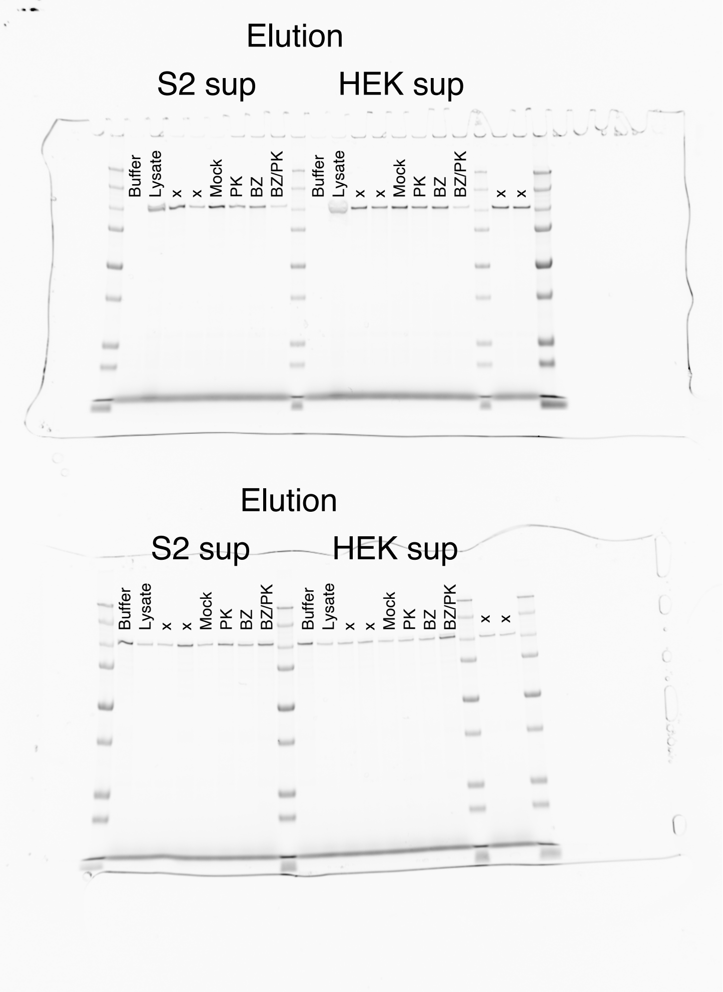

# Fig.1C raw image

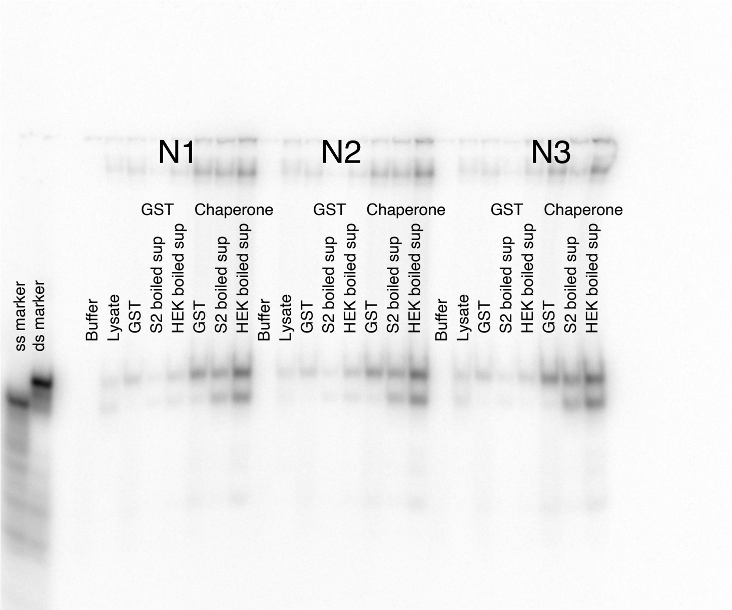

# Fig.3A raw image

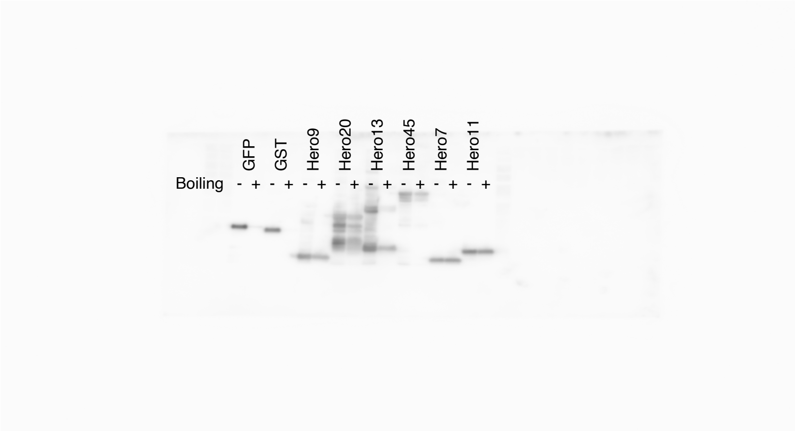

# Fig.4B raw image

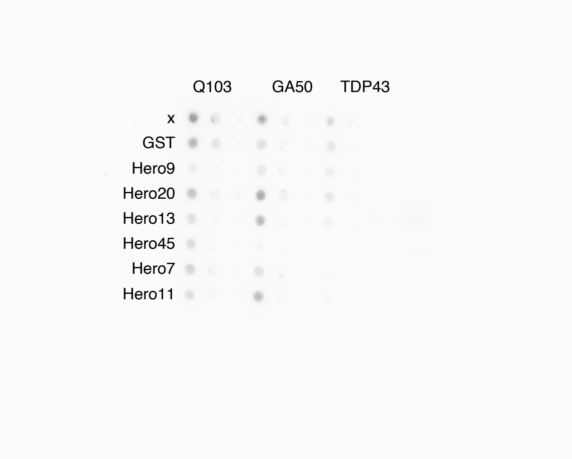

# Fig.4C raw image

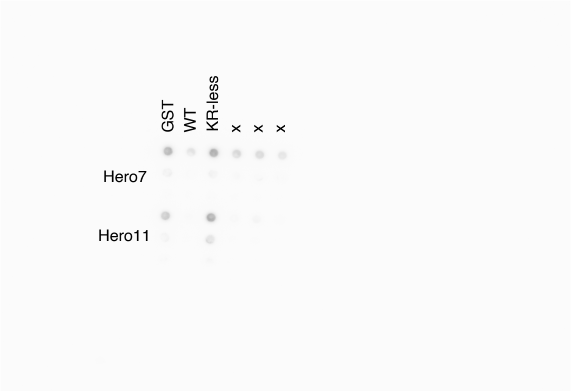

# Fig.4D raw image

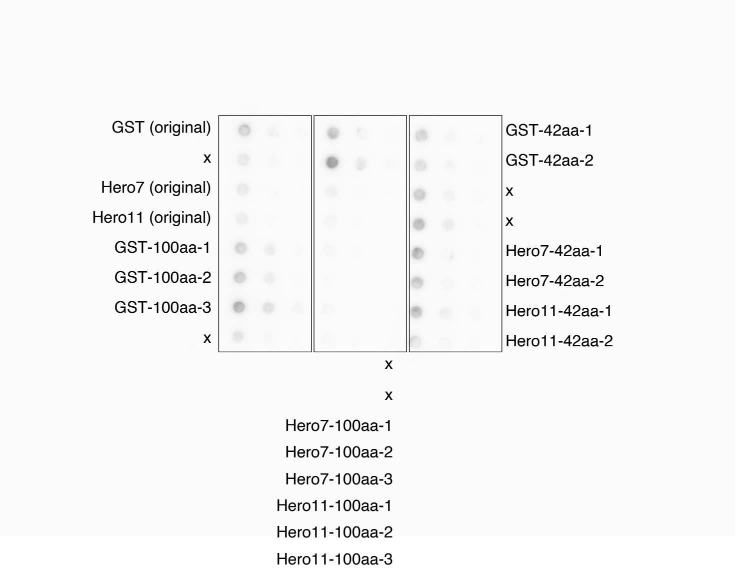

# S2 Fig raw image

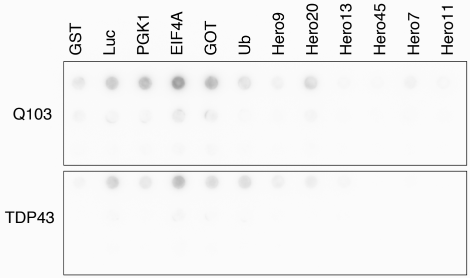

Supplement: S1 Raw Images — (PDF) [file pbio.3000632.s008.pdf]
