# Supplementary material for: A widespread family of heat-resistant obscure (Hero) proteins protect against protein instability and aggregation
Source: PLoS Biol. 2020 Mar 12;18(3):e3000632. doi: 10.1371/journal.pbio.3000632 (PMC7067378; doi:10.1371/journal.pbio.3000632)
Supplement: Attachment — Submitted filename: 191227_PbP.docx [file pbio.3000632.s009.docx]

**Point-by-point response to the Reviewers’ comments**

**Rev. 1:**

The manuscript submitted to PLoS Biology by Tsuboyama et al. reports on an intriguing group of heat resistant, charged, and disordered proteins that exhibit protective effects on a variety of protein substrates. They also exhibit positive effects in cells and even organisms on aggregation and aging, respectively. The impetus for the study was the existence of protective proteins in radically distinct organisms and by virtue of the fact that a fusion protein containing Ago2 could be resolubilized after incubation with lysate. After isolation by resistance to severe heat denaturation from S2 cells and human (HEK293) tissue culture cells, the heat resistant lysate resolubilized Ago2 and reactivated the Ago2-containing RISC complex and rescued a model protein from dehydration. The isolated activity was partially protein and nucleic acid dependent. Mass spectroscopy was then used to isolate the surprising large number of proteins in the lysates (>900 in both S2 and HEK cells), which led to a bioinformatic analysis that helped characterize their features: lack of hydrophobicity, a high score in a composite disorder prediction, and the presence of positive and negative charges.

Ultimately, the authors selected a group of “HERO” proteins exhibiting these properties. A subsequent analysis of the HERO proteins—individually—yielded fair to impressive effects (and even negative effects in some cases) in a range of assays/activities: protein aggregation, resistance to dehydration, and reactivation, as well as neuronal protein aggregation, neurite outgrowth, resistance to protein aggregation in the Drosophila eye, and Drosophila life span. Overall, an interesting collection of factors with a range of chaperone-like activities have been isolated through a careful analysis, one that encompasses a formidable amount of data.

We appreciate the Reviewer’s positive evaluation of our study.

Because the importance of this study relies not on the direct isolation of the HERO proteins but on the computational prediction of their existence, the study lacks a critical control: Instead of using GFP and GST as negative (arbitrary) controls, it was surprising that the investigators did not select proteins that were predicted, using their computational tools, to lack the desired properties inherent in the six selected HERO proteins. How might a collection of these negative controls behave in the assays employed? While this is not essential for every assay described in the paper, there are some experiments in which select HERO proteins are inactive, exhibit negative consequences, or at best exhibit ~2x improvements. How would a random collection of six proteins, or six proteins that lack the HERO attributes, behave in these assays? This is vital to buttress the central hypothesis of the study and to ensure that the observed effects do not reflect stochastic effects of the HERO proteins assayed.

We thank the Reviewer’s for raising this important point. According to the Reviewer’s suggestion, we have added several well-structured proteins as negative controls in our key experiment, i.e., the filter trap assay to monitor protein aggregation. We found that human PGK1, EIF4A1, GOT1 and Ubiquitin as well as firefly luciferase cannot suppress protein aggregations (now shown in Figure S2). This result supports our hypothesis that the fully unstructured property of Hero proteins is important for client protein stabilization.

Second, since the HERO proteins are evolutionarily diverse and are not related to previously identified LEA proteins, it is likely that they exhibit combinatorial effects. Have any pairs of the HEROs, or even the HERO collection, been examined? For the in vitro studies, this would be trivial, as subcritical concentrations—perhaps reflecting their cellular levels—could be combined with available preparations. For some of the cell-based studies, it would similarly be trivial to coexpress 2 or even 3 HERO proteins. Indeed, if the HERO proteins exhibit these protective functions in cells, they should work in combination to protect cellular proteostasis.

We are grateful to the Reviewer for his/her interest in our study. We fully agree with the Reviewer that diverse Hero proteins are likely to act synergistically to protect the cellular proteostasis in vivo. Given that there are hundreds of Hero protein candidates, each of which has different yet partially overlapping client preference, the combinatorial effects must be vast and complex. We are eager to tackle this promising direction in the future, but we anticipate that it will require a combination of more systematic and high-throughput methods than simple mixing or co-expression of arbitrarily chosen Hero proteins. Based on the discussion with the Editor, we would like to focus on characterization of individual Hero proteins in our current study, which should serve as a critical starting point toward the goal.

Other Comments:

1. In the cellular and organismal assays, the authors should discuss the potential that the HERO proteins rely on the activities of classical chaperones, many of which exhibit similar properties and endogenously expressed.

Indeed, it is possible that some Hero proteins rely on the activities of classical chaperones. Conversely, some classical chaperones may be assisted by the protective effect of Hero proteins. We have now discussed such possibilities in the text.

2. On p. 9, the authors propose that the disparate effects of the HERO proteins reflect distinct preferences for protein clients, but this does not explain why one HERO protein, SERF2/HERO7, exhibits pro-aggregation properties.

Purified Hero7/SERF2 has a tendency to phase separate under a low temperature in vitro, which may be responsible for the pro-aggregation property of this protein. Yet, Hero7 can effectively protect LDH from desiccation in vitro and luciferase from heat in cells. We humbly admit that, except for the suppression of TDP-43 aggregation, we still do not know the “rule” underlying the different client preference of different Hero proteins. We hope to obtain clues by utilizing more large-scale and comprehensive approaches in the future.

3. On p. 10, the authors refer to a previous study in which random IDPs were selected (Matsuo et al., 2018). But, what were the pI values for those IDPs examined in the study? Did they also exhibit a “bimodal distribution”?

Matsuo et al. used 6 short unstructured peptide fragments (35–45 aa), whose pI values are 5.31, 4.53, 4.16, 7.87, 6.24, and 5.05, respectively. Their client-protective activities were much weaker than those of (full-length) Hero proteins in our current study, presumably because of their short amino-acid lengths and narrower pI ranges.

**Rev. 2: Edward Wallace – please note that this reviewer has waived anonymity**

This manuscript identifies a class of protein chaperones that they call Heat-resistant obscure (Hero) proteins, that maintain their solubility in response to boiling, which chaperone the function of protein complexes (Ago2-RISC) without requiring ATP, and whose deletions give rise to phenotypes in drosophila and in human cell lines. The paper uses many different assays to carefully show the behaviour and importance of the Hero proteins. These observations are of major importance and wide interest, with potential applications as the authors discuss. It is also a cool story about how a “negative control” led to a new discovery.

The manuscript has already been through one round of review and the authors have responded thoroughly to those reviews, adding important and extensive new data. The work is clearly written, and of a high standard as well as of wide interest. I have three remaining concerns: first, the data availability is not acceptable for PLOS policies nor for community standards of FAIR science; second, biological replication is not clearly described; third, recent work on heat-denaturation of proteins at physiological temperatures needs to be addressed.

We thank Prof. Wallace for his encouraging and enthusiastic comments on our study.

Data availability

The manuscript’s Data Availability Statement that "All data supporting the findings of this study are available from the corresponding authors on reasonable request,” does not comply with PLOS policy that "PLOS journals require authors to make all data underlying the findings described in their manuscript fully available without restriction, with rare exception.” There is no foreseeable reason for exception here. Currently, the supplementary data provided are inadequate.

I urge the authors to make their data available in their own interests, because well-organized open data increases the community’s trust in manuscript findings and thus increases citations, and is the best defence against the sadly common event where authors themselves might lose track of the data in future years. I urge them to read carefully PLOS policies on data availability, and also the excellent guide from the Leek lab: <https://github.com/jtleek/datasharing>.

Practically speaking, the key data are the proteomics LC-MS/MS data whose analysis is summarized in Figure 2. Raw data must be deposited in an archive such as PRIDE. The PSM data used to make Figure 2 must be included, in full, in a supplementary data file. This should be easy as the plots were clearly made in ggplot2 from data in tabular format, and indeed now it is easy to share both data and plotting code on platforms such as figshare. At a minimum, for all samples/fractions/replicates of both the drosophila and human datasets, the supplemental data must include (a) PSM counts for every detected protein (Figs 2A/B), (b) a table indicating the group for every detected protein (x axis, Figs 2C-J).

Methods must be revised so as to conform with the minimum information about a proteomics experiment guidelines (MIAPE; Taylor et al., Nat Biotechnol. 2007).

We appreciate the Reviewer’s constructive suggestions. Accordingly, we have added the tables including the PSM count and the categorized group for every protein detected in LC-MS/MS (new Table S1 and S2). We have also made a substantial revision of Methods, which now conform with MIAPE-derived more strict criteria currently used by proteomics-specialized journals.

Replicates.
There is almost no mention of biological replicates or repeat experiments. Quantitative biochemical data, whether gels or proteomics, generally requires at least 2 independent biological replicates. The qualitative conclusions of the study are strong due to the many independent assays used that argue towards the same conclusions. For example, the proteomics data in two organisms and the gel/blot data presented overall agree that these obscure proteins are indeed heat-resistant. The recombinant FLAG-tag validation is clear including negative controls absent from the supernatant after boiling. The extensive functional validation of chaperone activity in vitro and in vivo is impressive. However the manuscript needs to state how many repeats there were, and acknowledge limitations in quantification as a result.

In the fly work, "Five female heads were quantified from each [eye cryosection] genotype.” How many flies were used in the other experiments, and how many replicate experiments?

We have now added all the information of biological replicates or repeat experiments, particularly in the fly work, in the respective Figure Legends or within the Figure (Fig. 7D). We have also included all the raw images of gels and blots and the quantified numerical data with replicates in Supporting Information.

Prior work on heat-denaturation (or not) of proteins

The abstract says "Proteins are typically denatured and aggregated by heat.” Then in introduction, “Proteins are generally vulnerable to heat, which destroys their structures and induces denaturation and aggregation.” Recent data counteract this long-established paradigm: importantly, Paola Picotti's lab published a careful proteome-wide stability analysis showing that many proteins are not denatured even far above physiological temperatures. Meanwhile, slight heat stress can cause specific protein aggregation that appears to be stress-protective rather than denaturation (reviewed in the citation by Franzmann and Alberti, as well as by Triandafillou et al., JBC 2019). Aggregation behaviour depends on both temperature and on factors such as pH and buffering conditions, as well as protein chaperones. The results statement "Many proteins are naturally unstable even under physiological conditions, probably because they need to prioritize function over structural durability,” could encompass this point that a protein's function could depend on its instability or aggregation.

The observations in this manuscript are new in that they reveal a class of proteins that maintain their solubility even at near-boiling temperatures very far above physiological temperature. Key points of the abstract and introduction should be moderated to reflect these recent findings and specify the range of temperatures involved.

We fully agree with the Reviewer that we should utilize more specific wordings like “near-boiling temperature” to characterize Hero proteins, instead of just “heat”. We have cited the paper from the Picotti’s lab and modified the abstract and the main text accordingly.

Minor comments

In figure 2, why are some panels boxplots and others violin plots? These are two different ways of showing distributions, wouldn’t it be clearer to have all box or all violin?

We intentionally utilized two types of graphs; box plots are suitable for indicating the median values as well as the 25th and 75th percentile, while violin plots can better show the overall distribution shape of the data. We wished to focus on the distribution in Figures 2C, D, I, J, K and L, while we wished to highlight the change of the median values in Figures 2E, F, G and H.

It is confusing that TDP is used for both tardigrade disordered proteins and for TDP-43, can this be fixed?

We fully agree with the Reviewer that “TDP” is somewhat confusing. However, TDP-43 is a well-established protein name (initially reported by Ou et al., J Virol 1995), while TDPs (tardigrade disordered proteins) was coined by Boothby and colleagues (Boothby et al., Mol Cell 2017). We do not think we are at liberty to rename them by ourselves.

Review response figures R1-R4 should be included as supplementary figures.

We thank the Reviewer for his interest in our preliminary data. Although promising, we are afraid that they are still too precursory to be included in a formal paper. We hope to solidify and publish these data in the future.
