# Supplementary material for: A widespread family of heat-resistant obscure (Hero) proteins protect against protein instability and aggregation
Source: PLoS Biol. 2020 Mar 12;18(3):e3000632. doi: 10.1371/journal.pbio.3000632 (PMC7067378; doi:10.1371/journal.pbio.3000632)
Supplement: Attachment — Submitted filename: PBIOLOGY-D-19-03113R2 PLOS Formatting Requests.docx [file pbio.3000632.s010.docx]

PLOS Formatting Requests

# For: “A widespread family of heat-resistant obscure (Hero) proteins protect against protein instability and aggregation”

To access your manuscript and complete these changes, please follow the link in the email and find your manuscript in the ‘Submissions Needing Revision’ folder on your main menu. Please also provide a tracked changes file indicating any updates.

**This is your FINAL opportunity to make amendments to the scientific content of your manuscript.** We strongly recommend that all authors double check ALL files prior to the final versions being submitted. Content changes requested at later stages WILL NOT BE ACCEPTED.

**Questions?** Email [plosbiology@plos.org](mailto:plosbiology@plos.org) quoting your manuscript number. We also recommend reading our [author submission guidelines.](http://journals.plos.org/plosbiology/s/submission-guidelines)

# METADATA CHECK

Please ensure that all information entered in the submission form is correct. This information will be indexed in PubMed and published by *PLOS Biology* as written.

- An **Early Version** of your manuscript:
  - WILL NOT be published online ahead of the final version

If you (or your institution) are planning a press release for this article, please contact us as soon as possible to ensure that an early version of your paper is not published.

- Please check the **authorship and affiliations** list on the title page of your manuscript. These details will be used to index the authorship of your manuscript and therefore should be checked carefully for any errors.
- Please ensure that if you have any protocols associated with [protocols.io](https://www.protocols.io/) in your manuscript, they are made public before publication to guarantee immediate access to the methodological details.

Please confirm that the following elements are correct and written in full sentences. Please update as needed.

- **Financial Disclosure**
- **Competing Interests**
- **Data Availability Statement**

# MANUSCRIPT REQUIREMENTS

Please update your manuscript with the following changes:

1. Please provide a detailed Financial Disclosure statement in the submission form. Include grant numbers and URLs as appropriate, as well as the initials of the authors who received the funding. This is published with the article, therefore it should be completed in full sentences and contain the exact wording you wish to be published.

Please add the statement 'The funders had no role in study design, data collection and analysis, decision to publish, or preparation of the manuscript' to the end of your Financial Disclosure statement on the submission form. If this statement is inaccurate, please describe the role the funders had.

If you received no funding, please add the statement 'The author(s) received no specific funding for this work' to your submission form.

OK

2. In your Data Availability Statement please ensure you have provided all necessary access information in complete sentences. This statement will appear on the published version of your manuscript.

OK

3. Please ensure your short title is correct in the submission form, including symbols, italicisation etc. Your short title will be typeset from the submission form directly.

OK

4. Please indicate the corresponding author on the title page with an asterisk (*) and include the corresponding author's email address. Only an asterisk and the email address itself are required.

OK

5. Please ensure that all authors have at least one affiliation listed on the title page and that all listed affiliations are attributed to at least one author. Affiliations should be listed in ascending numeric order in the affiliation list and should match the information provided in the Authors information in the submission form.

Affiliations should include an institution, a city, and a country (**no postcode**). US affiliations need the state as well, written out in full (**with no abbreviations**).

OK

6. Please remove the funding information from your manuscript file. The published version is pulled automatically from the Financial Disclosure section of the submission form.

OK

7. Please remove the Competing Interests section from your manuscript file. The published version is pulled automatically from the Competing Interests section of the submission form.

OK

8. Please remove your Author Contribution section from your manuscript file as this information is automatically pulled from the Authors information in the submission form.

OK

9. Please remove your Data Statement section from your manuscript file as this information is automatically pulled from the Data Availability question on the submission form.

OK

10. Please note that the acknowledgements section should be used to acknowledge only those who contributed explicitly in some way to the study but who do not meet the criteria for authorship. We respectfully ask that you remove the following: Please remove the funding information from the Acknowledgements section of your manuscript file. The published version is pulled automatically from the Financial Disclosure section of the submission form.

OK

11. Your references need to be formatted according to the ICMJE standard reference style. PLOS uses the numbered citation [citation-sequence] method and first six authors, et al. Authors should be listed as "Last name, First Initial". Please note that references should be cited in square [ ] brackets and not round ( ) brackets to ensure that they are correctly typeset. Additionally, the reference list should be formatted as “1.” and not “[1]”. Please adhere to the PLOS style for all references. For a full outline see our Author Guidelines: <http://journals.plos.org/plosbiology/s/submission-guidelines#loc-references>

OK

12. Please ensure that all supporting information file names use the following format verbatim in the file inventory. The file names themselves must use an underscore rather than a space, i.e. S1_Fig.tif, S1_Table.xlsx, S1_Text.doc, S1_Data.xlsx, S1_PRISMA_Checklist.docx, etc. Each file type should be numbered starting at 1. Please also make sure that the file description matches the file name (e.g. S1_Fig.tif is listed as S1 Fig).

OK

13. Please ensure that each supporting information file has a legend at the end of the manuscript file, after the Reference list.

OK

14. Please ensure that all supporting information legend titles use the following format verbatim in all in-text citations (including spelling and capitalization): S1 Fig, S1 Table, S1 Text, S1 Data, S1A Fig etc. Each file type should be numbered starting at 1.

OK

15. Please note that your submission cannot contain any references to generic "supporting information" files and instead must refer to specific files using our naming convention (S1 Fig, S1 Table, S1 Text, S1 Data, etc.)

    OK

If Supporting Information files are included with your article, note that these are not copyedited and will be published as they are submitted. Please ensure that these files are legible and of high quality (at least 300 dpi) in an easily accessible file format. For this reason, please be aware that any references listed in an SI file will not be indexed. For more information, see our [Supporting Information guidelines](http://journals.plos.org/plosbiology/s/supporting-information).

# FIGURE REQUIREMENTS

PLOS offers a figure-checking tool, PACE (<http://pacev2.apexcovantage.com/>), to help authors to ensure all figures meet PLOS requirements so that the quality of published figures is as high as possible. **Please use this tool to help you address the requests below.**

PACE is a digital diagnostic and conversion tool for figure files. It will provide information about any failed check(s) and, if able, will automatically convert the figure file into an acceptable file that passes quality checks. PACE requires you to register for an account to ensure your figure files are processed securely.

Please complete the following instructions regarding your figure files:

1. Please ensure that all main figure files follow PLOS naming conventions in the File Inventory. The file name should be in the format “Fig1.tif” and the file description should be “Fig 1”.

OK

2. Please ensure that all main figure files are cited in ascending numerical order in the main text of the article.

OK

3. Please ensure that all main figures are referenced in the manuscript as Fig 1, Fig 2, etc. (including capitalization), rather than Figure 1, Figure 2, etc. Please note, however, that the file names themselves must not include the space, i.e. Fig1.tif, Fig2.eps.

Please use the following examples of naming conventions for citing multiple figures in your manuscript:

- Figs 3 and 4

- Figs 3, 4, and 7

- Figs 1-5

- Fig 3A and 3B

- Figs 3A and 7B

OK

4. Place each main figure legend directly below the paragraph in which that figure is first referenced.

OK

5. Please send us all the original gels in individual files at the original resolution, as the resolution of the images in the combined PDF is very low.

OK

6. Please indicate in the raw gel of Fig. 4D which lanes correspond to Hero7 original and Hero11 original in the final figure.

OK

7. Please note that all figures and supporting information files will be published under the Creative Commons Attribution License (creativecommons.org/licenses/by/4.0/). Under this license authors retain ownership of the copyright for their article, but allow anyone to download, reuse, reprint, modify, distribute, and/or copy articles in PLOS journals for any reason, even commercially, so long as the original authors and source are cited.

   We ask that you please confirm that you either created the following figure panels or have written permission from the copyright holder to publish them under a CC-BY license:

   - illustrations in Figs 1, 3, 6 and 7

   OK. We are the original creator of the images.

   If you are not the original creator of the image, we do ask that you provide written confirmation via email from the copyright holder. Please note that any written confirmation you send to us must clarify that the copyright holder understands and agrees to the terms of this license.

Please note that this is your only chance to adjust figures, changes to figures (including resizing) at proof will not be accepted.

For a full outline, please see our [figure](http://journals.plos.org/plosbiology/s/figures) and [table](http://journals.plos.org/plosbiology/s/tables) preparation guidelines.

You can also upload a Striking Image to accompany your article if one is available. If this image is judged to be suitable, it may be featured on our website. Images should be high resolution, eye-catching, single panel images. If your image is from someone other than yourself, please ensure that the artist has read and agreed to the terms and conditions of the Creative Commons Attribution License. Note: we cannot publish copyrighted images.
